# Moving toward a common goal via cross-sector collaboration: lessons learned from SARS to COVID-19 in Singapore

**DOI:** 10.1186/s12992-022-00873-x

**Published:** 2022-09-21

**Authors:** Soojin Kim, Yuki Goh, Jun Hong Brandon Kang

**Affiliations:** Public Policy and Global Affairs Programme, School of Social Sciences, Nanyang Technological University, 48 Nanyang Avenue, HSS-05-02, Singapore, 639818 Singapore

**Keywords:** COVID-19, Health Crisis, Cross-Sector Collaboration, Partnerships, Singapore

## Abstract

**Background:**

The spread of COVID-19 has taken a toll on many countries and its healthcare system over the last two years. Governments have sought to mitigate the repercussions of the pandemic by implementing aggressive top-down control measures and introducing immense fiscal spending. Singapore is no exception to this trend. Owing to a whole-of-society approach, Singapore is still being lauded globally for its relatively successful record at controlling both community and trans-border spread. One notable effort by the Singapore government has taken place through its cross-sectoral collaborative partnerships with the private stakeholders behind the success.

**Methods/results:**

In an attempt to better explain Singapore’s robust yet strategic response to COVID-19, this study focuses on how the experience of the SARS outbreak has informed the government’s collaborative efforts with other stakeholders in society, beyond mere transnational cooperation. Taking a comparative case study approach in the specific context of Singapore, we perform a content analysis of related government documents, mainstream newspaper articles, and academic journal articles in an *inductive* manner. By closely comparing two global healthcare outbreaks, we note four differences in approach. First, during the COVID-19 pandemic, Singapore has focused on securing sufficient essential healthcare resources with contingency plans to strengthen preparedness. Second, the government has actively harnessed the capacity of private entities to promote the resilience of the healthcare system and the community. Third, Singapore’s management policies have been made not only in a top-down, centralized style during the initial response stage, but also with a greater proportion of bottom-up approaches, particularly as the pandemic trudges on. More interestingly, the multi-faceted repercussions of COVID-19 have gradually opened the door to a greater variety of collaborative partnerships in sectors *beyond* healthcare services. The participating stakeholders include, but are not limited to, local and international business actors, non-profit organizations, academia and other countries. Lastly, as the pandemic has continued, the Singapore government has managed *outward* to tap the expertise and knowledge of the private sector, in particular leveraging science and technology to improve control measures and putting supportive programs into practice.

**Conclusion:**

The evidence from our focused analyses demonstrates that the nature and scale of the COVID-19 pandemic produced more collaborative partnerships between the public and private sectors in Singapore as compared with the SARS outbreak. What is more, our findings offer evidence that through adaptive learning from the prior global healthcare outbreak, plus some trial and error during the initial phase of the ongoing pandemic, public- and private-sector partners, both in and outside of the healthcare service sector, have tended to “act alike,” working together to achieve a common goal. Both have been socially responsible, providing public services to people in need to promote the rapid resilience of the community, and sharing the associated risks. Overall, this study has deep and wide implications for other governments and policy makers who are still struggling to maximize essential resources and minimize the negative impacts of the healthcare crisis.

**Supplementary Information:**

The online version contains supplementary material available at 10.1186/s12992-022-00873-x.

## Introduction

Over the past two years, we have witnessed unprecedented impacts of the COVID-19 pandemic on global health and the global economy. First discovered in 2019 in Wuhan, China following a cluster of unidentified pneumonia cases, the novel coronavirus spread rapidly not only in Wuhan but also worldwide [69], and has since undergone several waves of mutations (such as the Delta and Omicron variants). As of August 10, 2022, more than 580 million COVID-19 cases have been reported around the globe. Cases peaked at over 3,800,000 in a single day in January 2022, and over 6 million people have died of COVID-19 [87]. As COVID-19 rages on, many healthcare systems worldwide are on the brink of collapse, in some cases due to a lack of medical facilities or professionals. Consequently, the estimated cost of the pandemic has added $24 trillion to the collective global debt as of February 2021, bringing it to a whopping $281 trillion [36].

Given this, recent scholarship has highlighted the importance of collaboration across all sectors of society as a way forward out of the pandemic and toward a more resilient healthcare system, a full economic recovery, and continued long-term growth (e.g., [12, 63, 80]. This is reminiscent of Bryson and colleagues’ (2015) definition of cross-sector collaboration as “the linking or sharing of information, resources, activities, and capabilities by organizations in two or more sectors to achieve jointly an outcome that could not be achieved by organizations in one sector separately” [10], p. 44 as cited in [11], p. 648). In this vein, it can reasonably be argued that by effectively mobilizing stakeholders and resources, multisector collaborative partnerships can help alleviate the strain on public finance, especially for governments, and can provide cost-efficiency gains and drive appropriate and satisfactory essential services to the most disadvantaged people [52, 66].

Over the past two years of the global health crisis, strategic multisectoral collaboration efforts have been put into practice in many developed countries, including the United States, Australia, Canada, and Japan, to ensure the timely and continuous delivery of essential goods and services, including medical assistance [66]. This is in line with the World Health Organization’s (WHO’s) Emergency Risk Management for Health (ERMH) which has identified multi-sectoral approach as a guiding principle for managing health crises [85]. These collaborations may take the form of a multi-ministry-centric hybrid organization that plays a leading role in preventing and controlling disease spread within a community by investing in sustainable diagnostic solutions and digital solutions to current and future needs. For this, diverse industries such as medicine, education, science and technology may produce these solutions together with policy support from the government.

Singapore is no exception to this trend. Unlike other Asian countries such as South Korea or Taiwan that faced upticks in COVID-19 cases after initially containing the spread of the virus, Singapore is still being lauded globally for its relatively successful record at controlling both community and trans-border spread. It has also maintained a lower mortality rate than much of the world [1]. Given its small geographic size and high population density, the city-state has adopted a so-called “a defensive pessimism stance,” remaining vigilant and preparing for worst-case scenarios [82]. Notably, during the COVID-19 pandemic, Singapore’s government has demonstrated its readiness and agility, taking more proactive and strategic action than it did during previous health crises (e.g., the Severe Acute Respiratory Syndrome (SARS) outbreak in 2003). The government’s initial policies to contain COVID-19 were “top-down” in style. It managed *downward* at the initial response stage, but since then has also managed *outward,* forming collaborative partnerships with other stakeholders over time, including the private healthcare and economic sectors (including local and international businesses), non-profit organizations, academia and other countries (e.g., see [42]).

Some scholarship has compared Singapore’s response to the COVID-19 pandemic with its response to the SARS outbreak in 2003, as both diseases were caused by coronaviruses.^1^ However, most research on Singapore’s success against COVID-19 has tended to describe chronological, medical data (e.g., the death toll); the country’s in-house capacity resulting from its enhanced healthcare system; its early response and disease surveillance efforts (e.g., [17, 47], or public sentiments about the related policies (e.g., [71, 82]. Little attention has been paid to cross-sector collaboration, particularly related to the role of private actors in and outside of the healthcare service arena. In response to this gap in the existing literature, this study aims to closely map the extent of the multisector collaboration efforts that have been implemented by Singapore’s government during the ongoing COVID-19 pandemic. In the focused analyses, we use Singapore’s experience during the SARS outbreak as a point of reference as we evaluate and discuss the progress made in governmental partnerships with other stakeholders.

## Background: the SARs and COVID-19 outbreaks in Singapore

Given that coronaviruses have been responsible for both outbreaks, this study posits that reviewing Singapore’s experience with SARS and COVID-19 and comparing the government’s response to the two outbreaks can reveal the adaptive learning that has taken place over time. Table 1 provides some basic details as to how Singapore was affected by the SARS and COVID-19 global health crises.

**Table 1:** Comparison between the SARS and COVID-19 Outbreaks in Singapore

	**SARS**	**COVID-19**
**Outbreak period (Global)**	November 2002 to July 2003	November 2019 to present
**Outbreak period (Singapore)**	March 2003 to June 2003	January 2020 to present
**First reported case in Singapore**	March 1, 2003	January 23, 2020
**Total confirmed cases**	238	1,773,386
**Local Death toll** **(Case fatality rate)**	33 (14%)	1543 (0. 09%)
**Rapid diagnostics**	RT-PCR/reverse transcription polymerase reaction	RT-PCR/reverse transcription polymerase reaction/Antigen-rapid test (ART)
**Medical prevention**	Not available (No FDA-approved vaccination)	Available (FDA-approved vaccines and antiviral pills)
**Governance Structure**	From the Operations Group (Led by the MOH) to the three-tiered taskforce (Led by the MHA): Inter-Minister Committee (IMC), Core Executive Group (CEG) and Inter-Ministerial SARS Operation Committee (IMOC)	Multi-Ministry Taskforce (MTF) (Currently led by the Minister for Health and the Minister for Finance together)

In the case of COVID-19 outbreak, total confirmed cases and local death toll were calculated as of August 10, 2022

*Source*: Compiled from various sources [39, 49]

### The SARS outbreak in 2003

In November 2002, the first ever reported case of SARS occurred in Foshan, China, when patients presented clinical flu-like symptoms such as high fever with unexplained pneumonia. However, China did not proceed with state-level control measures nor report the cases to the World Health Organization until February 11, 2003 [45]. The virus later spread to 29 other countries including Singapore [35]. Globally, 8422 confirmed and probable cases were reported. A total of 916 deaths from SARS were reported by the end of the pandemic in June 2003 [14].

Singapore recorded its first case of SARS on March 1, 2003, when a traveler returning from Hong Kong was admitted into Singapore’s Tan Tock Seng Hospital (TTSH). Her case was linked to a superspreading event at Metropole Hotel [18]. Singapore soon saw an exponential increase in cases following outbreaks in several public settings such as hospitals [24]. Crucially, 41% of the cases were healthcare workers due to the multiple hospital-related outbreaks that took place at TTSH, National University Hospital (NUH), and Singapore General Hospital (SGH) [18, 35]. Singapore became one of the top 5 most affected countries, recording a total of 238 cases and 33 deaths, resulting in a case fatality rate of 14% [57]. The epidemic ended in Singapore in June 2003 after the WHO declared Singapore SARS-free on May 30, 2003.

**Fig. 1:**
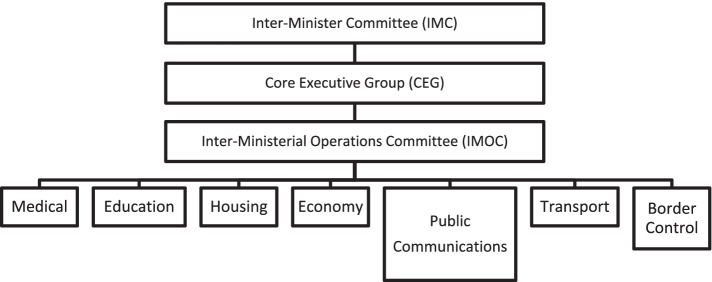
SARS Governance Structure. Source: Adapted from Tay & Mui (2004, p. 35) [76]

The SARS outbreak was initially and directly managed by Singapore’s Ministry of Health (MOH). The Operations Group by the MOH, announced in the middle of March 2003, was led by the Director of Medical Services and comprised on senior doctors and administrators from various public hospitals. The Group was in control of all the medical resources and served as the link between the MOH and all healthcare providers [39]. But later, as the government realized that resources from other ministries were required to combat the outbreak, a three-tiered national control structure was created to strongly control the spread of the virus. As shown in Fig. 1, the three tiers included the Inter-Ministerial Committee (IMC), the Core Executive Group (CEG) and the Inter-Ministerial SARS Operation Committee (IMOC) [76]. The IMC was chaired by the Minister for Home Affairs and comprised of ministers from other ministries, including the MOH, the Ministry for Education (MOE and the Ministry for National Development (MND. This committee served to: (i develop strategic decisions to design policies,(ii approve major decisions; and (iii implement control measures [39]. Meanwhile, the CEG was led by the Permanent Secretary for Home Affairs and directed valuable resources at the ministry level to key areas during the outbreak, whereas the IMOC carried out the health control measures issued by the IMC, coordinating interactions between the MOH and healthcare providers and frontline workers [39, 76].

### The COVID-19 pandemic from 2020 to the present

COVID-19 emerged in Wuhan, China in November 2019 and was reported to the WHO on the last day of 2019 [16]. On March 11, 2020, the WHO officially declared COVID-19 a pandemic. The virus has spread to more than 150 countries around the world. COVID-19 has similar clinical features to the flu, but the incubation period for COVID-19 before symptoms are developed may be as long as 14 days. These features have made the virus difficult to contain, and as of August 10, 2022, the WHO has reported close to 580 million confirmed cases and 6,418,958 deaths globally [86].

The first case in Singapore was reported on January 23, 2020 when a 66-year-old Wuhan resident tested positive for COVID-19 while traveling in Singapore [26]. Singapore has experienced multiple outbreaks over the last two years of COVID-19. The first major outbreak occurred in dormitories for foreign workers and accounted for the spike in cases early in April 2020. This led to a nationwide lockdown known as “the circuit breaker,” which lasted from April 7 to June 1, 2020. The circuit breaker largely contained the initial outbreak within the foreign workers’ dormitories and minimized the spread to the community. However, Singapore experienced two more massive outbreaks in the community in the latter half of 2020/first half of 2021 and in the first half of 2022, which were primarily attributed to the spread of the Delta and Omicron variants, respectively [2]. As of August 10, 2022, Singapore had recorded a total of 1,773,386 cases and 1543 fatalities [61]. The case fatality rate is therefore around 0.09%, much lower than the rate with SARS cases (See Table 1).

**Fig. 2:**
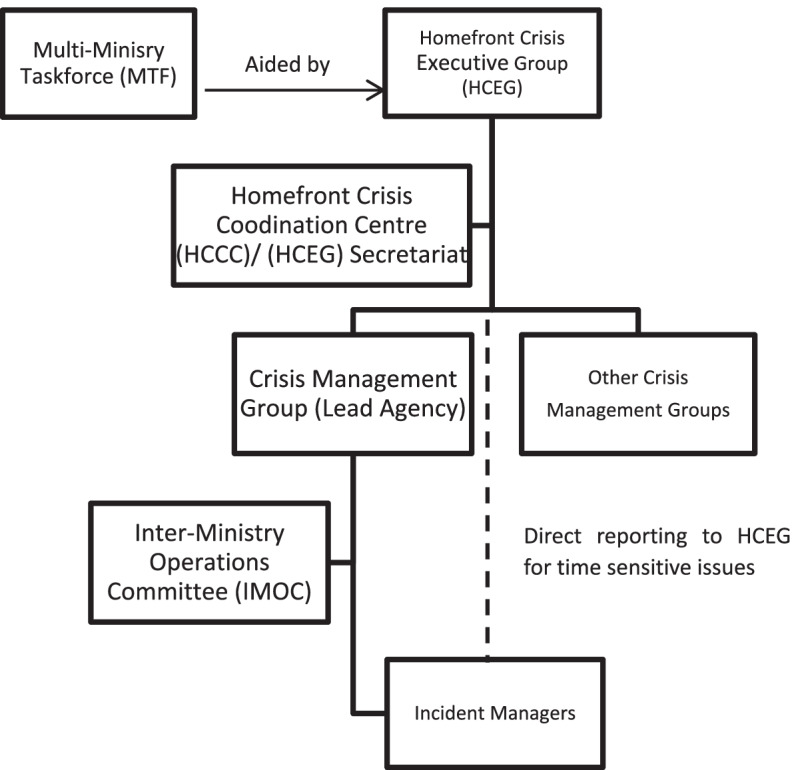
COVID-19 Governance Structure. Source: Adapted from Low (2020) [50]

In terms of the government decision-making of health emergencies, the Singapore government established an inter-departmental organization named the Homefront Crisis Executive Group (HCEG) prior to 2004. HCEG is chaired by the Permanent Secretary of the Ministry of Home Affairs (MHA) and comprises senior representatives from all ministries but the major role of the HCEG was to provide the strategic and political guidance during the health crisis (e.g., the endorsement of MOH’s recommendations for the suitable Disease Outbreak Response System Condition (DORSCON)^2^ level). During the COVID-19 pandemic, although essential medical resources are mainly controlled by the MOH, there was an urgent need to coordinate health (control) measures across governmental sectors as well as across the complete society. In turn, to facilitate inter-departmental communication in setting priorities into action, the government has become to set up a Multi-Ministry Taskforce (MTF) which was jointly led by the Minister for Health and the Minister for National Development in the initial phase [1]. Interestingly, the number of ministries participating in the MTF has been more than doubled compare to the SARS outbreak. The MTF on COVID-19 consists of the Ministry of Communication and Information (MCI), the Ministry of Trade and Industry (MTI), the Ministry of the Environment and Water Resources (MEWR), the National Trade Union Congress (NTUC), the Ministry of Education (MOE), the Ministry of Manpower (MOM), the Ministry of Social and Family Development (MSF), and the Ministry of Transport (MOT) [41]. The main tasks of the MTF have been not only to direct the national whole-of-government response to COVID-19 outbreak, but also to work with the international community to respond to the outbreak. For example, this taskforce has focused on border controls, the circuit breaker (lockdown), and addressing the outbreak in the migrant worker dormitories. In addition, in order to continue to prevent the public from underestimating the risks of COVID-19 and the mitigation policies from backfiring, the MTF has tried to coordinate the community response by carrying out weekly press conferences to convey critical information (e.g., daily contact-tracing reports or stepwise criteria for reopening the economy) to the public [23, 82]. All in all, the MTF has actively supported the HCEG to effectively deliver their plans and decisions to the elected leadership for political direction and ensure subsequently confirmed actions to be taken in practice (See Fig. 2) [50, 51].

## Methods and analyses

During the SARS outbreak and the ongoing COVID-19 pandemic, the Singapore government has embarked on multiple service delivery projects in collaboration with private actors to manage, pre-empt and mitigate challenges associated with the outbreaks. In order to scrutinize and compare Singapore’s response to the two global healthcare crises, this study uses *inductive* conventional content analysis based on secondary data obtained through major local newspaper articles, related government agency and industry reports, and academic journal articles describing how Singapore responded to each outbreak. Specifically, as with Berg and Lune [9] and Hsieh and Shannon [32], we attempted to identify and cluster evidence-based themes derived directly from the text data in a document and then discover hidden meaning of the related content.

Building on Baxter and Casady’s [12] so-called recovery framework model, we discuss the Singapore government’s specific milestones related to multisectoral collaborative partnerships during the two global health crises (See Tables 2 and 3 below; for more detailed information, see Appendices A and B). It should be noted that while Baxter and Casady [12] focused on healthcare policies alone, our analysis expanded the model to include non-healthcare policies.

**Table 2:** SARS: Multisector Collaboration

	**Healthcare**	**Non-Healthcare**
**Short-term,** **Reactive** **Response**	• Diversion of non-flu cases away from TTSH to be handled by general practitioners (GPs) • Development of Infrared Thermal Fever Scanner by ST Electronics with DSTA	
**Medium-term,** **Proactive** **Response**	• Bilateral arrangements with Malaysia and Indonesia to facilitate contact-tracing and quarantine • Joint Declaration of the Special ASEAN Leaders Meeting on SARS 2003	• Monitoring of employees’ temperatures by major hoteliers in cooperation with the Singapore Tourism Board (STB) • STB launch of ‘Cool Singapore Awards’ to acknowledge major hoteliers and tourist facilities

**Table 3:** COVID-19 Multi-Sector Collaboration

	**Healthcare**	**Non-Healthcare**
**Short-term,** **Reactive** **Response**	• Public Health Preparedness Clinics (PHPCs) were activated • Private hospitals cared for well and stable COVID-19 patients • Masks were produced locally by Innosparks and ST Engineering • Surbana Jurong constructed Community Care Facilities (CCFs) • Staff from private hospitals provided medical care in CCFs • TvVax, made up of healthcare professionals from the private sector, secured vaccines for the population • Duke-NUS medical school developed serological test kits to boost contact-tracing efforts	• Temasek Foundation provided reusable masks and hand sanitizers • Migrant Workers Center and Alliance for Guest Workers Outreach delivered food and provided support for migrant workers in isolation • VisualAid provided cards with translations of healthcare terms to improve healthcare workers’ communication with migrant workers • Joint statement of ASEAN Defence Ministers on Defence Cooperation against Disease Outbreak (ASEAN, 2020) • Grab offered GrabCare catered to the transport needs of healthcare workers
**Medium-term,** **Proactive** **Response**	• Hotels were converted into isolation and quarantine facilities • SIA and DHL were tasked with the handling and delivery of vaccines • Private healthcare providers ran the vaccination centers set up by the MOH • Ramatex worked with A*STAR to develop more effective masks suited for Singapore’s climate	• Private firms provided training and partially funded trainees’ allowance under SG United Traineeship program • Ministry of Health, SG United, and Nanyang Polytechnic worked with GovTech Singapore together to improve the effectiveness of a new app called *TraceTogether*
**Long-term,** **Future-oriented** **Response**	• Private telemedicine providers offered enhanced home recovery programs • ASEAN Strategic Framework for Public Health Emergencies and ASEAN Regional Reserve of Medical Supplies	

**Fig. 3:**
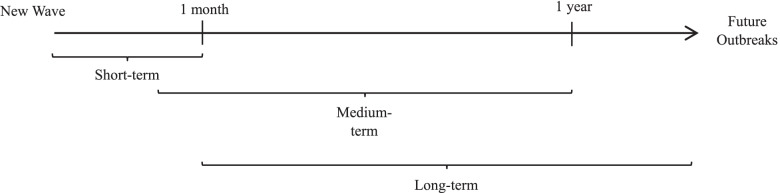
Relative Timeframes of Healthcare Crisis Responses. Source: Authors modified Baxter and Casady's (2020) PPP-based analytical approach

As seen in Fig. 3, the responses of a government facing a healthcare crisis can be divided into three different broad timeframes. In terms of short-term response, a government is expected to respond promptly to an existing outbreak or an unanticipated new wave of a disease. Such reactive responses are likely to emerge between the start of a new wave of the outbreak and about one month into an outbreak. Crisis-driven government-led collaborative management is meant to meet the immediate needs of the healthcare system and society. In the medium term, responses are more proactive and are expected to emerge anywhere from a few weeks after the initial outbreak to one year into the outbreak. During this period, cross-sector collaboration can help governments anticipate the challenges of the pandemic. The responses may include product development or the strengthening of existing healthcare services. Long-term responses are those aimed at pre-empting future outbreaks, and they require careful planning for the future based on adaptive learning. It is expected that these responses will emerge from one month after the initial phase of virus transmission to beyond a year, depending on when an outbreak ends and how serious its transmission is. In the post-outbreak period, the government may consider continuing its collaborative partnerships with actors including private firms,^3^ non-governmental organizations (NGOs), academia and even international organizations of a transnational nature to strengthen the existing healthcare system and other service industries and reduce the severity of future pandemics.

## Results

### Short-term reactive response

In response to the extreme uncertainty brought about by a global health crisis, as mentioned above, governments initially tend to behave reactively to meet the immediate demands not only of the healthcare system, but also of the community. In practice, as seen in Tables 2 and 3, collaborative partnerships among public agencies and/or between the public and private sectors can meet pressing needs of a community in a state of agitation (e.g., by ensuring the resilience of the food and essential medical equipment/devices supply chains) [12].

In the early stages of the SARS outbreak, the Singapore government was not adequately prepared to deal with the infectious disease that was fluid and unprecedented [39]. Due to the initial lack of dedicated testing and isolation facilities and on-going contact-tracing effort, the first patient who had contracted SARS was only hospitalized and isolated after five days upon her return from Hong Kong, during which she had spread the virus to 22 other individuals [24]. Given this, the MOH initiated a SARS taskforce to study and manage the unexpected spread of the virus throughout the community, focusing on collaboration within the public sector that could better serve the public [35]. However, in practice, the presence of the first confirmed SARS cases within one public hospital––Tan Tock Seng Hospital (TTSH)––highly influenced the condition of other patients in the same hospital and caused the further spread of SARS to other healthcare institutions such as Singapore General Hospital and National University Hospital [24]. In response, to effectively contain the spread among various healthcare workers, institutions, and patients, the government tapped into the so-called “all-in-one” approach which has been considered as a unique Singapore term. In turn, at first, TTSH was designated as a SARS hospital by the MOH on March 22, 2003 [35, 39]. That is, the “all-in-one” approach required all suspected and confirmed cases of SARS were sent only to TTSH. Restrictions were also imposed on the movement of healthcare workers and patients among hospitals [35]. Meanwhile, all elective procedures in other public hospitals were placed on hold as the MOH redirected non-flu illness cases (that is, non-SARS related emergency patients) to other public hospitals and local general practitioners (GPs) [39]. The Singapore Armed Forces (SAF) were also activated to provide manpower support to public hospitals [54]. All these efforts were intended to prevent the overstraining of hospitals and to prevent cross-contamination.

In addition, during the SARS outbreak, to curb trans-border spread of the disease, a mandatory health declaration was imposed on travelers to Singapore. The Defence Science and Technology Agency (DSTA) under the Ministry of Defence collaborated with Singapore Technology (ST) Electronics to develop a temperature scanner, the Infrared Thermal Fever Scanner (Defence Science & Technology Agency, 2003) [21]. This cross-sectoral collaboration, which capitalized on the thermal imagers commonly used in the military, led to the rapid deployment of the scanner within one week at immigration checkpoints at Changi Airport to prevent the entry of suspected SARS patients [44].

During the COVID-19 pandemic, the Singapore government has moved in a timely way to roll out preventive strategies intended to control the spread of the disease in the community, across borders, and in hospitals. Notably, there has been an increase in collaborative partnerships to meet the needs of the healthcare industry as well as society—the general populace, healthcare workers and foreign workers—and such partnerships have been found not only in the healthcare arena but also in non-healthcare fields (e.g., IT, R&D, and the economic sector). As indicated in Table 3, for example, as the first line of defense, Public Health Preparedness Clinics (PHPCs) were activated in February 2020, within a few weeks of the first COVID-19 case in Singapore. GPs enrolled in the PHPC scheme underwent courses on the importance of infection control and were trained on the use of personal protective equipment (PPE) [15]. Because patients with respiratory illnesses were offered subsidized treatment at PHPCs, these patients were diverted away from hospitals to clinics, and only suspected positive cases of COVID-19 were referred to hospitals for further diagnostics [29]. Additionally, in contrast to Singapore’s SARs response, in March 2020, private hospitals in the city-state were allowed to collaborate with public ones to better accommodate stable patients, preventing a hospital bed crunch and ensuring that public hospitals had enough capacity to deal with more severe cases of COVID-19 [19].

Despite these timely response efforts, within a few months, confirmed positive cases of COVID-19 in local communities had increased substantially, and Singapore’s government faced the simultaneous challenge of a massive outbreak of new cases in the foreign workers’ dormitories where daily cases reached the thousands [1, 89]. This was largely attributable to the cramped living arrangements therein and residents’ lack of access to protective supplies such as masks and hand sanitizers [89]. In response, the Singapore government required foreign workers’ dormitories to be isolated and to undergo mass testing. What is more, to ensure that patients including foreign workers in critical condition received immediate attention and treatment with enough medical manpower, the government engaged Surbana Jurong, a private consultancy firm, to convert exhibition centers into Community Care Facilities (CCFs). The CCFs were used to accommodate individuals with mild symptoms that it is not required to have extensive medical treatment [51]. These were similar to the temporary hospitals, such as Huoshenshan Hospital, that were constructed in a matter of days in China. Given Surbana Jurong’s expertise and networks in the construction industry, they were well equipped to overcome the logistical issues posed by disruptions to the supply chain [51]. Meanwhile, the medical care in CCFs was provided by personnel deployed from private hospitals [51].

The imposition of mandatory wearing of face masks in Singapore coincided with a massive supply chain shortage as countries that were major producers of such masks were in lockdown and people worldwide were scrambling for masks. The Ministry of Trade and Industry (MTI) initially distributed masks from their stockpiles, but this was unsustainable. The Temasek Foundation, a subsidiary of Temasek Holdings, later became the main distributor of masks and other precautionary items such as hand sanitizers in Singapore [78]. Given Temasek Holdings’ broad networks due to its diverse investment portfolio on every continent, it was able to procure reusable masks that were new innovations and of better quality [79]. Some of the masks distributed utilized technology by Swiss-based Livinguard and UK-based DET30. Temasek Holdings’ distribution of essential items ensured that the MTI could divert their resources to the procurement of other items such as food while ensuring that the populace received better quality masks [50].

Apart from the Temasek Foundation’s procurement of masks from overseas suppliers, there have also been local efforts to restart the domestic production of masks amid worries of future supply chain shortages as the demand for masks increases globally. The domestic production of surgical masks, overseen by the MTI, was aided by Innosparks at ST Engineering, which had experience producing N95 masks [4]. These masks were meant to be distributed to healthcare workers amidst a market shortage of medical-grade masks. Meanwhile, the shortage of masks meant for the general public was also addressed by private firms such as Razer, which set up an automated manufacturing line that has been able to produce up to 5 million masks per month (CNA, 2020b) [20].

Furthermore, the government noted the importance of securing vaccines early on to reduce the death toll and curb the spread of COVID-19. This led to the formation of a Therapeutics and Vaccines expert panel (TxVax) that included 18 scientists and clinicians across hospitals, research groups, and the private sector in April 2020 [30, 83]. While the approval of vaccines was eventually done by the Health Sciences Authority (HSA) like a normal medication approval process, the panel played an additional yet a critical role in recommending the more promising vaccines directly and swiftly to government planners and the MOH for early procurement logistics after examining and discussing the results of the clinical trials of prospective vaccines [83]. In addition to increasing the healthcare sector’s capacity and procuring vaccines, there were also efforts to improve contact-tracing, which was the bedrock of containment of the disease in its initial phases. The MOH engaged a research team from Duke-NUS Medical School to conduct serological tests during the early phases of COVID-19, in which serological tests were limited [20]. Serological testing allowed for the detection of past infections even after an individual had recovered, allowing for more precise contact-tracing [65]. In practice, the development of the test helped contact-tracers detect the source of a cluster of 23 COVID-19 cases in the initial phase of the epidemic in Singapore and stemmed further outbreaks in the community [65]. The incident was the world’s first successful use of the serological test kit [65].

Aside from healthcare policies, multi-sector collaboration on non-healthcare policies was intended to meet other needs of society. During the outbreak in the foreign workers’ dormitories which led to the isolation or hospitalization of many foreign workers in CCFs, NGOs such as Healthserve, Transient Workers Count Too (TWC2), Singapore Migrant Friends, and the Alliance of Guest Workers Outreach worked with the inter-agency task force to cater meals suited to the tastes of foreign workers and provided psychological support to those in isolation [13, 67, 81]. Additionally, given the language barrier between local healthcare workers and foreign workers, a volunteer project, VisualAid, was also rolled out to provide informational cards containing terms translated into six different languages to help healthcare workers communicate more effectively with foreign workers [43].

Healthcare workers meanwhile faced discrimination from the general public while using public transport due to the public’s fear of contracting the mysterious new virus [74]. Such discrimination resulted in difficulties for healthcare workers looking for a ride home from hospitals after their shifts. Grab, one of the top-ranked mobile app–based private transport service companies in Southeast Asia, stepped in to resolve the challenge by launching GrabCare. GrabCare is similar to the company’s ride-hailing services, but caters specifically to healthcare workers traveling to and from their workplaces with the fixed fare for all 24 h, and employs only those drivers who voluntarily sign up for the service.

Aside from domestic multisector collaboration, the Singapore government has also signaled its commitment to transnational collaboration at the Association of Southeast Asian Nations’ (ASEAN’s) Defence Ministers’ meeting (ADMM) on February 19, 2020 where the management of COVID-19 was discussed. The joint statement issued on Defence Cooperation against Disease Outbreak emphasized the importance of information sharing to facilitate domestic contact-tracing and quarantine efforts [6].

### Mid-term proactive response

In line with Baxter and Casady’s [12] typology of short-term, medium-term, and long-term governmental responses, we note that medium-term partnerships between the public and private sectors represent a shift away from reactive responses to proactive responses and anticipation of potential challenges in a pandemic. Multisectoral collaborative partnerships may, for example, facilitate product development, strengthen existing healthcare services, or repurpose existing facilities to improve society’s resilience to potential outbreaks. But notably, during the COVID-19 pandemic, such partnerships have progressively expanded into non-healthcare arenas, given the economic repercussions a prolonged pandemic can have on society. In short, governments can use this strategy to work toward an economic recovery, thereby further stabilizing and strengthening the economy against the backdrop of an ongoing pandemic.

Because the SARS outbreak was over in 3 months, it resulted in minimal healthcare partnerships. Nevertheless, the outbreak had longer-term economic repercussions in Singapore, particularly for the tourism industry. The Ministry of Trade and Industry (MTI) reduced its GDP growth forecast from 3% to 0.5% after the initial outbreak of SARS [40, 55]. In addition, the unemployment rate reached a peak of 4.8% (higher than during the 2007–2009 Global Financial Crisis) for a few months after the end of the SARS outbreak in September 2003 [68]. These signals of an economic downturn prompted the government to work with the private sector to revitalize the economy and reduce retrenchment. For instance, as seen in Table 2, one notable initiative was the collaboration between the public sector Singapore Tourism Board (STB) and major private sector hoteliers. While Singapore’s borders remained partially open to travelers, foreigners were wary of visiting Singapore due to the rapid spread of the virus and the country’s strict quarantine orders. Singaporeans were also reluctant to staycation at hotels and instead chose to stay home to avoid contracting the disease during that period. This situation led to the collaborative partnership between the STB and hoteliers to provide travelers and Singaporeans assurance that hotels were safe environments by monitoring the temperatures of hotel employees [31]. The initiative later expanded into a full-fledged certification system known as the ‘Cool Singapore Awards,’ which were awarded to hotels and other tourist attractions. The certification worked as a motivator to participants to ensure their complete adherence to government health advisories and the disinfection of their facilities during the SARS outbreak [31].

Aside from its efforts to prop up the local economy, the Singapore government also engaged in transnational cooperation to mitigate the cross-border spread of SARS. For instance, beginning with bilateral arrangements with neighboring countries such as Malaysia and Indonesia, Singapore has sought to exchange the information required for contact-tracing and quarantine to ensure that visitors are safe [35]. Later, through the Joint Declaration of the Special Meeting by ASEAN Leaders on SARS 2003 and an ASEAN + 3 summit involving ASEAN leaders, China, Japan and South Korea, Singapore further collaborated with other countries to facilitate information-sharing and pre-departure screenings to reduce the cross-border transmission of SARS [31].

The longer duration of the COVID-19 pandemic has illustrated the need to increase the resilience of the healthcare system to battle the next outbreak while also ensuring that the economy recovers. The summer of 2020 saw massive outbreaks on every continent while Singapore was barely able to control the spread in foreign workers’ dormitories [90]. The need for stronger measures to mitigate community outbreaks that could bog down the healthcare infrastructure and to reduce mortality rates led to closer partnerships between the government and hotels. One of the control measures was an issuance of stay-home notice (SHN),^4^ a form of individual quarantine orders for all travelers. The government prevented travelers and returning Singaporeans from completing SHN at their place of residence in order to prevent household spread, but for the measure to be effective, more dedicated SHN facilities were needed. More than half of the hotel rooms in Singapore were put to this use through July 2020 [77]. While some hotels have reopened to accommodate staycationing Singaporeans, these hotels remain ready to be converted back to quarantine facilities if required [88].

In addition to expanding Singapore’s healthcare facilities, the private sector has also made significant contributions to Singapore’s vaccine roll out. In a bid to achieve herd immunity by vaccinating the population as quickly as possible, the Singapore government started its national vaccination drive in January 2021, accompanied by rigorous public outreach and media coverage (e.g., TV and radio spots, personal SMS from the MOH, social media campaigns, and printed brochures). In addition, the government prepared financial assistance and insurance packages (e.g., on-time pay-out) for cases with serious side effects. Given the temperature-sensitive nature of the vaccines, air transportation business partners including Singapore Airlines (SIA) and DHL Global Forwarding played a critical role in delivering the vaccines from overseas [91].^5^ As a result, Singapore became the first Asian country to receive the Pfizer-BioNTech shots from Brussels, Belgium in December 2020, and Moderna’s COVID-19 vaccines arrived in Singapore in February 2021 once they were approved by the government.

Even after the delivery of the vaccines, the vaccination programs were conducted jointly by the two sectors—the MOH and private medical providers. Community roll-out of the first doses of the vaccines started on February 22, 2021 [59]. The government aimed to complete COVID-19 vaccinations by the third quarter of 2021 to keep the virus under control.^6^ It strongly encouraged residents to get the jab and first made vaccines available to Singapore Armed Forces personnel, then workers in the land transport sector, seniors aged 70 and above, seniors aged 60 to 69, and each progressively younger age group in a timely sequence. In order to ensure the seamless and efficient roll-out of vaccines as planned, the MOH set up 36 vaccination centers including public general hospitals (e.g., for frontline healthcare workers), community centers, and 10 mobile vaccination teams island-wide. The tender to run these vaccination centers, worth a total of $38 million, was awarded to 17 healthcare providers in February 2021 [92]. The main service providers from the private sector have been Raffles Medical and Fullerton Health. Although the vaccination dosage interval was initially increased from 6 to 8 weeks due to a supply crunch, it was later shortened to 4 weeks to ensure that the population could be vaccinated quickly [60].

**Fig. 4:**
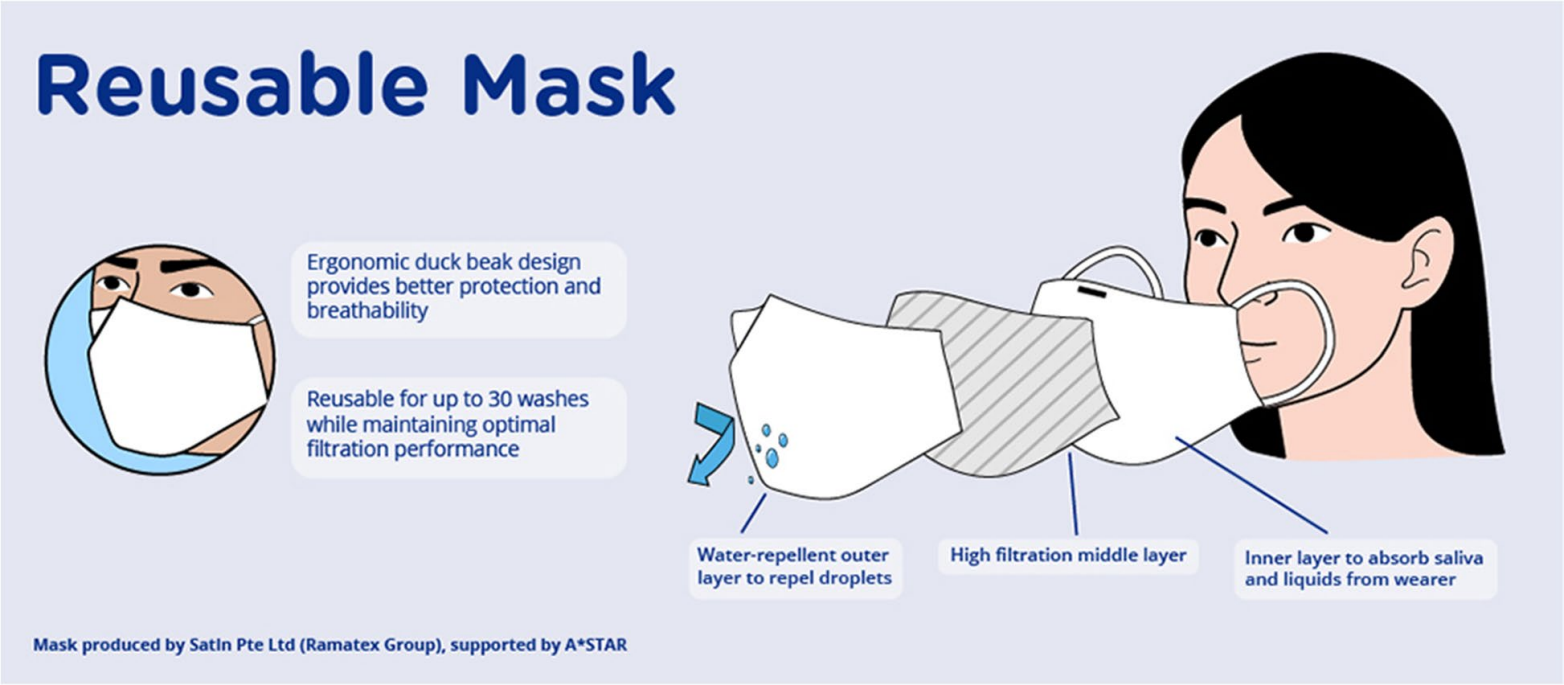
Details of mask produced by Ramatex and A*STAR. Source: A*STAR [3]

The local production of masks in the short-term was accompanied by ongoing innovations to increase the efficiency of production lines. In particular, the Agency for Science, Technology and Research (A*STAR) collaborated with Ramatex to design more effective masks [4]. Through the combination of A*STAR’s scientific knowledge and Ramatex’s expertise in textiles, the collaboration was able to produce a reusable mask that was as effective as medical masks, as shown in Fig. 4.

Aside from increasing the resilience of the healthcare sector, the government also sought to reduce youth unemployment brought about by the pandemic through the SG United Traineeship program, which works with private organizations to provide traineeships to recent graduates. Participating companies ranged from financial institutions such as DBS Bank, to telecommunications and event management firms such as Singtel and Kingsmen, respectively. As part of the program, Workforce Singapore (WSG), a government agency (a statutory board) under the Ministry of Manpower, has funded about 80% of the training fees while the participating private companies have agreed to pay the remaining costs.

Another major impact of COVID-19 was the closure of borders, which was extremely detrimental to Singapore’s small and open economy. Singapore’s government has continued to strive to open its borders safely once pandemic situations in its key partners stabilize. One key pillar in this initiative is to ensure that travelers are vaccinated or test negative for COVID-19 pre-departure. To facilitate information sharing about vaccination status, technologies such as Israel’s Green Pass and the European Covid Digital Certificate have been used. Building on the Open Attestation Framework developed by the Government Technology Agency (GovTech), private companies such as Accredify and Trybe.ID helped to amplify the reach of HealthCerts in other countries through their international networks [28]. The private companies have therefore enabled HealthCerts to be used in 9 countries and 420 medical facilities [28]. Foreign buy-in to HealthCerts for the storage of digital records of COVID-19 tests has been critical to the reopening of borders in Singapore and elsewhere.

Another instance of Singapore’s government capitalizing on digitalization is the collaboration between the MOH, SG United, GovTech Singapore and Nanyang Polytechnic to develop and further enhance the effectiveness of an app called *TraceTogether* across different models of phones [72]. The collaboration capitalized on the latter two’s existing facilities to accurately measure the signal strength between two phones [42].

### Long-term future prevention-oriented response

According to Baxter and Casady [12], in the long-term, governments may continue their multisectoral partnerships and/or trans-national partnerships to ensure sufficient service delivery to the people. Yet it should be noted that relative to COVID-19, the short timeframe of SARS reduced the possibilities for long-term cross-sector collaboration. In contrast, in its response to COVID-19, Singapore’s government has shifted from a pandemic response to an endemic one, suggesting that it intends to maintain and expand such collaborative partnerships. Domestic policies that emphasize living with the virus, as we do with influenza, and eventually easing control measures, can be considered a long-term, strategic response to COVID-19.

Singapore’s population is highly vaccinated, with 90% having received the full regimen as of March 2022. Thus the government has increasingly strived to transition toward treating COVID-19 as endemic [70]. As with influenza or dengue, when COVID-19 is considered endemic, occasional outbreaks will be expected, but a shift will be seen to home recovery over hospitalization [37]. Given that the virus is still prevalent in many other countries and that there are wide disparities in vaccination rates internationally, the Singapore government has needed to ensure that healthcare policies and related infrastructure are in place for home-based treatment and focused care. For example, private telemedicine enterprises such as CommCare, Doctor Anywhere, Fullerton Health, and HiDoc were brought in to reinforce the MOH’s home recovery program by providing virtual consultation to COVID-19 patients including children. Their services have also included delivery of medications and in-person swabbing that can accommodate home recovery. Later, general practitioners (GPs and dentists in a voluntary manner have stepped into this telemedicine care for their own patients [62, 75].

In addition, looking to the future, Singapore has actively sought to prevent or at least mitigate the impacts of a future global pandemic by building up its international cooperation and collaboration chains. One notable effort by the government has taken place through the ASEAN member states. The ASEAN Strategic Framework for Public Health Emergencies was finalized in late 2020. It provides for a multilateral approach to public health emergencies and increases the capacity of ASEAN’s public health networks [56]. In particular, an ASEAN Regional Reserve of Medical Supplies was created to enhance the region’s ability to stockpile essential medical items such as PPE to protect healthcare workers and prevent a shortage as seen during the initial phase of the COVID-19 outbreak, when countries had to scramble to obtain PPE and oxygen ventilators [7].

## Discussion: evolution of collaborative partnerships from SARS to COVID-19

Although Singapore was out of the woods within a few months of the outbreak of the SARS virus, given that SARS was the first major communicable disease challenge of its kind, the government took away critical lessons that informed its policy responses to the COVID-19 pandemic [73]. Singapore maintained the key tenets learned from SARS and also managed to fight COVID-19 more effectively, not only by increasing its cross-sector collaborations, but also by mobilizing partnerships with private entities such as NGOs, academia, and neighboring governments and expanding these beyond the traditional arenas of the healthcare and economic sectors. The following four emerging themes demonstrate how Singapore learned from the prior outbreak and has responded differently to the recent global healthcare emergency.

### Ensuring sufficient essential healthcare resources and developing contingency plans

In order to manage a health emergency effectively, it is important to maintain access to and ensure sufficient infrastructure and logistics, including the stockpiling and distribution of healthcare resources and the establishment of temporary medical facilities [85]. One of the key takeaways of the SARS outbreak for Singapore was the importance of having excess capacity and contingency plans during a healthcare crisis, such as available hospital beds and medical resources for emergency cases (e.g., infected patients) to receive professional attention immediately [24, 84]. When the COVID-19 pandemic began, just as TTSH had been designated as the public hospital for SARS patients, the Singapore government swiftly confirmed the National Centre for Infectious Diseases (NCID), equipped with high-level isolation units, its own in-house laboratories, and technological features of a wearable tag-based real-time locating system for contact-tracing among healthcare workers, patients, and visitors, as the main hospital to treat patients who were critically ill with COVID-19 [33]. Given the general underlying expectation that healthcare resources can be easily overwhelmed in the short term due to the transnational nature (spread) of the infectious disease via human-to-human contact, the special designation of NCID by the government was a crisis prevention strategy to reduce the burden that had been placed on public hospitals such as TTSH during the SARS outbreak and to strengthen preparedness via medical-capacity building that could be activated during another SARS-like crisis [84].

### Harnessing private sector capacity to develop a whole-of-society response

Interestingly, beginning in the middle phase of the COVID-19 pandemic, the reliance on the NCID alone turned out to be insufficient to withstand several sharp spikes in local COVID-19 transmission and subsequent surges in hospitalizations. Thus, to rapidly strengthen its operational capacity and to reduce further strain on public hospitals, Singapore’s government started to develop collaborative partnerships with private hospitals in a whole-of-society approach [38, 84]. For instance, through the activation of the Public Health Preparedness Clinic (PHPC) scheme, the MOH encouraged private clinics and hospitals to work as government partners to help patients with emergency health needs and provide financial and material support (e.g., medications, swab tests, vaccines and professional training for healthcare workers). The establishment of PHPCs was in line with ERMH’s recommendations for governments to make advanced arrangements with private companies to ensure access to medical facilities during a health emergency [85]. As a result, acting as the first line of defense, PHPC-affiliated private actors have played an important role in reducing the burden on the operational capacities of public hospitals dealing with unconfirmed cases of COVID-19 [48].

Along with transferring medically stable COVID-19 patients to private hospitals for continued recovery, another notable strategy in the healthcare arena geared at accommodating more patients and providing enhanced medical support within a short time was to increase the number of newly-built CCFs, especially massive-scale facilities (e.g., about 10,000 beds at the Singapore EXPO and Changi Exhibition Centre) staffed with private healthcare workers and even volunteers [27, 51, 58]. This represents a fundamental change from Singapore’s SARS response in that the MOH showed reluctance to use private hospitals to fight the virus when SARS hit Singapore hard in 2003. Indeed, it suggests that the Singapore government has made extensive efforts to enable the continuous deliverance of healthcare services even as demand increased to a great extent. This was to strengthen and sustain the resilience of public health security of communities.

In a similar vein, there was a shift in quarantine practices from the SARS outbreak to the COVID-19 pandemic. During the SARS outbreak, individuals identified as close contacts of sick individuals served out their quarantine at home with minimal private resources utilized, except when enforced by private security agencies [35]. In contrast, during the COVID-19 pandemic, a large volume of private resources have been employed, particularly in the form of government-designated facilities such as affiliated hotels and makeshift convention halls operated by private organizations. Food providers have also played a peripheral role assisting with isolation and quarantine orders [58].

Moreover, the dramatic shortage of face masks during the initial outbreak of COVID-19 made clear the importance of procuring essential resources. The government initially released 5 million masks from its stockpile to retailers, but these were snapped up in a matter of a few hours by zealous Singaporeans [25]. Given the high demand for masks and the export bans in major mask-production countries like Taiwan, the Singapore government turned to domestic mask production, which had been non-existent, as well as relying on private procurement by Temasek Holdings.

Lastly, following breakthroughs in vaccine research, Singapore’s government aimed to vaccinate the entire population against COVID-19 as quickly as possible. As noted earlier, private sector healthcare organizations played a leading role in operating vaccination facilities across the region and providing vaccine services to the public.

### Allowing more bottom-up approaches together with top-down approaches

During the SARS outbreak, Singapore’s government managed *downward,* using top-down approaches to directly manage the public health crisis. However, during the COVID-19 pandemic, these government-led top-down approaches have been accompanied by society-driven bottom-up (ground-up) approaches involving collective actions and public–private partnerships. More notably, in an effort to achieve a common goal to return Singapore to normal conditions, through tapping on the bottom-up approaches, the government could embrace a variety of private actors and volunteers *beyond* the healthcare service sector.

For example, as noted earlier, one crucial bottom-up approach was the GrabCare initiative led by Grab, a private transportation and food delivery company, to offer dedicated rides for healthcare professionals who faced discrimination on other forms of transportation [8, 74]. This discrimination had the potential to lower the morale of frontline healthcare workers, putting greater strain on the healthcare system as healthcare workers battled mental health problems [53]. As large clusters began to rapidly form in a community and many people feared such evolving local transmission scenarios, the Singapore government’s ability to address this discrimination was limited, since a behavioral change among residents to show social acceptance of healthcare workers (e.g., avoiding the use of public transport or ride-sharing) seemed to involve a substantial commitment of time and effort. In turn, a bottom-up approach was more suitable. By the end of February 2020, about 50% of Grab’s driver pool had joined the program [74]. While Grab’s actions did not ignite the behavioral change required to eliminate discrimination against healthcare workers, it can reasonably be argued that the initiative has helped resolve the challenge, at least during the initial peak of the pandemic, ensuring that essential healthcare workers have been able to carry out their duties efficiently.

It is also notable that the Singapore government has formed partnerships with NGOs to help migrant workers, something that did not occur during the SARS outbreak. NGOs that frequently interacted with foreign workers provided expertise at responding to their particular circumstances and needs during the COVID-19 pandemic (e.g., by providing translation services to facilitate communication or catering food that would appeal to this population) [67]. For instance, VisualAid was another prominent bottom-up approach that occurred during the height of the outbreak in the foreign workers’ dormitories. The volunteer project was aimed at improving communication between healthcare workers and foreign workers, a topic of secondary importance to the government relative to ensuring that foreign workers were treated promptly and that the outbreak was stemmed. VisualAid created information cards with translations of medical terms, which were distributed to healthcare workers to help them communicate with foreign workers [43]. Indeed, the collaboration between NGOs and the government’s inter-agency taskforce ensured that foreign workers, who were not explicitly included in the national crisis response plan, received equal treatment and essential services for their healthcare and welfare [13].

### Leveraging science, research and digital technology

Taking a long-term view, as COVID-19 shifts from pandemic to endemic in Singapore, the government might be prepared to cope with uncertainties as future public-health outbreaks unfold. Arguably, as COVID-19 becomes endemic, more patients will likely be required to recover at home to prevent excess strain on the healthcare system. Given this expectation, in conjunction with the home recovery program, the government’s partnerships with private telemedicine providers could have made a difference. Specifically, they could help ensure that patients receive the primary care they need at home and even in quarantine without needing to seek treatment at hospitals, for example, via virtual consultation by the healthcare professionals and supervised self-swab COVID-19 Antigen Rapid Test (ART) over video call [46, 62, 75].

Not only did academia and the tech industry accelerate innovations based on their pre-existing knowledge in specific areas, these sectors also helped develop diagnostic solutions, contact-tracing, and vaccination status digitalization with the help of their state-of-the-art facilities and information technology (IT). For example, as mentioned earlier, the joint development of the cPass™ test kit by A*STAR and Duke-NUS Medical School shows how multi-sector collaboration can produce results quickly based on participants’ existing troves of knowledge and the research circulating in the academic sector [22. In addition, the collaboration between the MOH, SG United, GovTech Singapore and Nanyang Polytechnic helped ensure community-wide surveillance via the development of apps such as *TraceTogether,* even in the initial phase of the COVID-19 outbreak [72]. Further, during the mid-term phase of the pandemic, the Singapore government continued to use digital transformation to improve the government’s COVID-19 response [42]. One notable example is the cross-sectoral collaborative partnerships between GovTech and private organizations including Accredify and Trybe.ID to develop health passports that could ascertain the validity of travelers’ pre-screening details such as their vaccination status or the results of their COVID-19 tests, allowing Singapore to reopen its borders safely.

## Conclusion

In an attempt to better explain Singapore’s whole-government approach to tackling the spread of COVID-19, this study has focused on how the experience of the SARS outbreak has informed the government’s collaborative efforts with other stakeholders in society, beyond mere transnational cooperation. Taking a comparative case study approach, we performed a content analysis of related government documents, mainstream newspaper articles, and journal articles in an *inductive* manner. We were able to closely compare two global healthcare outbreaks and note both importance differences in their contexts and the government’s progress in terms of its more robust response to COVID-19.

The evidence from our focused analyses demonstrates that the nature and scale of the COVID-19 pandemic produced more collaborative partnerships between the public and private sectors in Singapore as compared with the SARS outbreak. First, during the COVID-19 pandemic, the government has focused on securing sufficient essential healthcare resources with contingency plans to strengthen preparedness. Second, the government has actively harnessed the capacity of private entities (e.g., private healthcare providers and manufacturers of medical products) to promote the resilience of the healthcare system and the community. Third, the government has proceeded with control measures and related management policies not only in a top-down, centralized style, especially during the initial response stage, but also with a greater proportion of bottom-up approaches, particularly as COVID-19 cases have continued to rage on in the community. In other words, the COVID-19 pandemic sparked more government-led collaborative partnerships and further led the government to embrace the ideas of community-based organizations. Notably, most collaborations led by private actors (e.g., GrabCare and VisualAid) have been voluntary, and their underlying goals have been to help vulnerable and at-risk groups (e.g., foreign workers and front-line healthcare workers). It can be argued that so-called community-based private organizations have played a fundamental role in alleviating the government’s burden through strong collaborative partnerships that have provided integrated care for the community (Yi et al., 2021) [89]. More interestingly, the multi-faceted repercussions of COVID-19 have gradually opened the door to a greater variety of collaborative partnerships in sectors *beyond* healthcare services. The participating stakeholders include, but are not limited to, the private healthcare and economic sectors (such as local and international business actors), non-profit organizations, academia and other countries [42]. Lastly, as the pandemic has continued, the Singapore government has managed *outward* to tap the expertise and knowledge of the private sector (e.g., its R&D capacity), in particular leveraging science and technology to improve control measures and putting supportive programs into practice (e.g., social distancing, diagnostic solutions and the digitalization of vaccination records) [23].

This paper makes several contributions to the literature. First, in the specific context of Singapore, which has been globally applauded for its successful control of the spread of COVID-19, comparing the government’s management of the SARS outbreak with its management of COVID-19 has allowed us to delineate what the government learned and how cross-sector collaboration expanded during the current pandemic. Second, this study provides a practical, chronological analysis of the implementation of related policy prescriptions to combat the pandemic, with a particular focus on how COVID-19’s larger scale has brought about an evolution in cross-sector collaboration since SARS (also see Appendices A and B). Specifically, by categorizing the various collaboration efforts into healthcare and non-healthcare service areas, and by closely examining Singapore’s response during three different timeframes (here, the short-term reactive response, the mid-term proactive response, and the long-term future-oriented response), this study has provided a detailed discussion of the topic. In addition, our findings offer evidence that through adaptive learning from the prior global healthcare outbreak, plus some trial and error during the initial phase of the ongoing pandemic, public- and private-sector partners, both in and outside of the healthcare service sector, have tended to “act alike,” working together to achieve a common goal. Both have been socially responsible, providing public services to people in need to promote the rapid resilience of the community, and sharing the associated risks.

As the war against COVID-19 continues around the world, Singapore’s strategic response against the pandemic can serve as a point of reference for other like-minded nations to cultivate a sustainable and effective long-term response against the pandemic. The lessons learned in Singapore have proved that as the short-term reactive response, public health measures implemented by the government alone could be effective, but they turned out to be unsustainable, especially as the world prepares to deal with an endemic disease like COVID-19. Collaboration between the government agencies and a variety of private actors thus can help prevent our public healthcare system from being overwhelmed and ensure that the public continues to benefit from higher quality of healthcare services in a sustainable and feasible manner. Given this, other governments and policy makers who are still struggling to maximize essential resources and minimize the negative impacts of the healthcare crisis may need to consider adjusting their response stance from managing *downward* to inviting more participation from private-sector entities and capitalizing on innovation to gain wisdom from their expertise and knowledge. At the same time, through closely working with voluntary organizations and civil society groups in a community, a nation’s social security net can be further complemented especially in times of crisis.

One limitation of this study is that it focuses only on the Singapore context, and readers should bear this in mind and take care when generalizing its results. Notably, Singapore is a small-sized city state in which neither subnational structures (e.g., federal-state relations) nor the rural-versus-urban continuum exist and its political system has been known as a competitive authoritarian state or an illiberal democracy [1, 64]. These characteristics could result in a more straightforward management method during SARS and COVID-19, relative to other countries. However, policies with different characteristics including varying fiscal capacity and different political and cultural environments may not diffuse along the same lines. Given this, we believe the findings of this study can provide a point of comparison for future work (e.g., cross-national case studies). In other words, future studies may continue to uncover additional points of evidence (practices) in different contexts. This is especially so given that the widespread and flexible cross-sector collaboration that have sprung up during the pandemic in various countries are likely to ignite better ways of collaboration in the future [63]. We also expect that collective planning and action will continue even in the post–COVID-19 era through partnerships across various public and private organizations. Thus, it may be worthwhile for focused research to employ more varied interpretive approaches, including survey questionnaires or focus group interviews with healthcare providers and workers.

## Supplementary Information

**Additional file1: Appendix A.** Timeline of Collaborative SARS Response. **Appendix B.** Timeline of Collaborative COVID-19 Response.

## Data Availability

All items reviewed are publicly available on-line. The datasets created and/or analysed during the current study are available from the corresponding author on reasonable request.

## Abbreviations

A*STAR: Agency for Science, Technology and Research, Singapore
ADMM: ASEAN’s Defence Ministers’ Meeting
ART: Antigen-Rapid Test
ASEAN: Association of Southeast Asian Nations
CCFs: Community Care Facilities
CEG: Core Executive Group
CNA: Channel News Asia
DSTA: Defence Science and Technology Agency, Singapore
EG: Executive Group
FDA: Food and Drug Administration
GDP: Gross Domestic Product
GLCs: Government-Linked Companies
GovTech: Government Technology Agency, Singapore
GP: General Practitioner
HCEG: Homefront Crisis Executive Group
IMC: Inter-Minister Committee
IMOC: Inter-Ministerial SARS Operation Committee
IT: Information Technology
MCI: Ministry of Communication and Information, Singapore
MEWR: Ministry of the Environment and Water Resources, Singapore
MHA: Ministry of Home Affairs, Singapore
MND: Ministry of National Development, Singapore
MOE: Ministry of Education, Singapore
MOH: Ministry of Health, Singapore
MOM: Ministry of Manpower, Singapore
MOT: Ministry of Transport, Singapore
MSF: Ministry of Social and Family Development, Singapore
MTF: Multi-Ministry Taskforce
MTI: Ministry of Trade and Industry, Singapore
NCID: National Centre for Infectious Diseases
NGO: Non-Governmental Organization
NTUC: National Trade Union Congress, Singapore
NUH: National University Hospital, Singapore
PHPC: Public Health Preparedness Clinic
PPE: Personal Protective Equipment
R&D: Research and Development
RT-PCR: Reverse Transcriptase Polymerase Chain Reaction
SAF: Singapore Armed Forces
SARS: Severe Acute Respiratory Syndrome
SGH: Singapore General Hospital
SHN: Stay-Home Notice
SIA: Singapore Airlines
ST Electronics: Singapore Technology Electronics
STB: Singapore Tourism Board
TTSH: Tan Tock Seng Hospital
TWC2: Transient Workers Count Too
TxVax: Therapeutics and Vaccines expert panel
WHO: World Health Organization
WSG: Workforce Singapore

## Footnotes

1 The last time the world witnessed an influenza pandemic on the scale of COVID-19 was the Spanish Flu of 1918. Yet, in the existing research, COVID-19 has more often been compared to the 2003 SARS outbreak, since both diseases emerged in the modern era and were caused by viruses in the *coronaviridae* family. In addition, given that the SARS outbreak was the first epidemic since Singapore became a developed country, as with Chen et al. [18], this study posits that SARS is the optimal case for comparison.

2 This is a color-coded framework that highlights Singapore’s  health crisis (disease) condition. There are four colors—green (the lowest), yellow, orange, and red (the highest) that represent each alert level of Singapore’s pandemic preparedness plan depending on the level (nature) of disease [1].

3 Given that government-linked companies (GLCs) in Singapore such as Temasek Holdings and Singapore Airlines are subject to the discipline of the stock market and investors, it can reasonably be argued that they function in line with a profit-maximizing objective, thereby acting more like private firms than state-owned enterprises [5]. Thus, this study treats GLCs as private entities.

4 The SHN is a legally binding document promulgated under the Infectious Diseases Act that requires travelers to remain in a dedicated facility for the stipulated duration. Failure to do so may be punishable under the Singapore law [34].

5 While DHL ensured that the cargo was successfully delivered from vaccine production facilities overseas to airports, SIA was tasked with ensuring that aircraft carriers were equipped with temperature-monitoring capabilities and cool boxes to transport the vaccines.

6 Covid-19 vaccines have been free for all Singaporeans and long-term residents, including those on an employment or S-pass, as well as work permit holders, foreign domestic workers, and holders of dependent passes, long-term visit passes, and student passes.
