# Supplementary material for: Moving toward a common goal via cross-sector collaboration: lessons learned from SARS to COVID-19 in Singapore
Source: Global Health. 2022 Sep 21;18:82. doi: 10.1186/s12992-022-00873-x (PMC9490717; doi:10.1186/s12992-022-00873-x)
Supplement: Supplementary file 1 — Additional file1: Appendix A. Timeline of Collaborative SARS Response. Appendix B. Timeline of Collaborative COVID-19 Response. [file 12992_2022_873_MOESM1_ESM.docx]

**­Appendix A. ­­Timeline of Collaborative SARS Response**

**Appendix B. Timeline of Collaborative COVID-19 Response**
